# The clinical picture and outcome in adults

**DOI:** 10.1186/1546-0096-12-S1-I16

**Published:** 2014-09-17

**Authors:** Dirk Elewaut

**Affiliations:** 1 Rheumatology, UZ GENT, Gent, Belgium

## 

The field of spondyloarthritis (SpA) has undergone substantial changes in the past decade. Increasing emphasis has been made on early diagnosis. Thus, MRI imaging has permitted new imaging modalities to detect early signs of inflammation in the axial skeleton. Additionally, much new data have emerged on the link between spinal inflammation and bone new formation, another hallmark of SpA. Classification of disease has evolved as well by focusing on the main clinical presentation (axial versus peripheral disease) and permitted to classify early forms of SpA. Early recognition facilitated early treatment regimens which showed markedly better responses in early disease and in patients with objective signs of inflammation (MRI positivity/ elevated CRP). Furthermore, there is increasing evidence that extra-articular manifestations of disease, particular gut involvement have marked impact on disease severity and long term outcome in SpA. It can be foreseen that extensive patient phenotyping will become critically important in the near future when new treatment modalities become available with differential efficacy on the various clinical features of SpA.

## Disclosure of interest

None declared.

